# Response of a Stoichiometrically Imbalanced Ecosystem to Manipulation of Nutrient Supplies and Ratios

**DOI:** 10.1371/journal.pone.0123949

**Published:** 2015-04-16

**Authors:** Zarraz M. Lee, Laura Steger, Jessica R. Corman, Marc Neveu, Amisha T. Poret-Peterson, Valeria Souza, James J. Elser

**Affiliations:** 1 School of Life Sciences, Arizona State University, Tempe, Arizona, United States of America; 2 School of Earth and Space Exploration, Arizona State University, Tempe, Arizona, United States of America; 3 Departamento de Ecología Evolutiva, Instituto de Ecología, Universidad Nacional Autónoma de México, Coyoacán, México D. F., México; University of Wisconsin Milwaukee, UNITED STATES

## Abstract

Cuatro Ciénegas Basin (CCB) is a desert ecosystem that hosts a large diversity of water bodies. Many surface waters in this basin have imbalanced nitrogen (N) to phosphorus (P) stoichiometry (total N:P > 100 by atoms), where P is likely to be a limiting nutrient. To investigate the effects of nutrient stoichiometry on planktonic and sediment ecosystem components and processes, we conducted a replicated *in situ* mesocosm experiment in Lagunita, a shallow pond located in the southwest region of the basin. Inorganic N and P were periodically added to mesocosms under three different N:P regimes (P only, N:P = 16 and N:P = 75) while the control mesocosms were left unamended. After three weeks of fertilization, more than two thirds of the applied P was immobilized into seston or sediment. The rapid uptake of P significantly decreased biomass C:P and N:P ratios, supporting the hypothesis that Lagunita is P-limited. Meanwhile, simultaneous N and P enrichment significantly enhanced planktonic growth, increasing total planktonic biomass by more than 2-fold compared to the unenriched control. With up to 76% of added N sequestered into the seston, it is suspected that the Lagunita microbial community also experienced strong N-limitation. However, when N and P were applied at N:P = 75, the microbes remained in a P-limitation state as in the untreated control. Two weeks after the last fertilizer application, seston C:P and N:P ratios returned to initial levels but chlorophyll *a* and seston C concentrations remained elevated. Additionally, no P release from the sediment was observed in the fertilized mesocosms. Overall, this study provides evidence that Lagunita is highly sensitive to nutrient perturbation because the biota is primarily P-limited and experiences a secondary N-limitation despite its high TN:TP ratio. This study serves as a strong basis to justify the need for protection of CCB ecosystems and other low-nutrient microbe-dominated systems from anthropogenic inputs of both N and P.

## Introduction

Nutrient limitation is widespread in both aquatic and terrestrial ecosystems [1], playing a major role in shaping their structure and function. Due to the scarcity of aquatic habitats in the arid environments, lakes and ponds are also key components of the ecosystem to the local community [2]. In arid environments, low nutrient lakes and ponds provide a large number of ecosystem services, including the major source of drinking water provision, recreational amenities and vital processes such as groundwater recharge and nutrient cycling. These freshwater ecosystems can also serve as sentinels to climate change [3]. However, many natural waters are increasingly vulnerable to nutrient perturbation from non-point source pollution as anthropogenic activities expand and intensify. Even relatively remote freshwater systems can experience increased nutrient loading and subsequent eutrophication via atmospheric deposition [4, 5]. Nutrient perturbation is of particular concern for such ecosystems because of the threats that eutrophication may impose on their unique biodiversity.

Restoration efforts of eutrophied lakes from diffuse pollution sources can be challenging and slow [6, 7]. Hence, it is important to understand how nutrient perturbation influences various aquatic habitats to better manage eutrophication and to evaluate time scales for recovery. Elucidation of responses to nutrient perturbation is further complicated by the interacting, and still intensely debated, roles of nitrogen (N) and phosphorus (P) in driving and maintaining eutrophication [8, 9]. P fertilization is often suggested to induce increased N inputs by nitrogen fixation; hence, decreasing P input is a primary focus to reduce eutrophication [10]. However, the potential for nitrogen fixation to offset N limitation is dependent on the planktonic community structure (N-fixers vs. denitrifiers) and energy available for this energy-demanding process [8, 11]. A meta-analysis of short-term bioassays in freshwater system found that N and P co-limitation is more widespread than single nutrient limitation and, at broad scales, the extent of short-term N or P limitation is similar across freshwater ecosystems [1]. Given these continued uncertainties and ecosystem-specific responses, it is unclear how the N:P ratio of inputs affect aquatic ecosystems with highly imbalanced N:P stoichiometry (i.e., where TN:TP ratios are often > 100). It is expected that ecosystems with high TN:TP ratio will be extremely P-limited and less likely to be affected by any changes in N loading.

Another important factor affecting the impacts of nutrient loading on the eutrophication process is water depth [12]. In particular, the effects of nutrient perturbation on the water column of shallow lakes and ponds are strongly influenced by sediment chemistry due to high sediment surface:water column ratios [13]. The sediment surfaces of shallow ponds in arid regions are often within the photic zone, providing optimal conditions for benthic primary production and biological assimilation of nutrients from the water column. The ability of sediments to buffer external nutrient load varies with the chemical and biological nature of the sediment [13]. For example, inorganic phosphate can be selectively sequestered via abiotic co-precipitation and adsorption processes depending on pH and calcium ions concentration [14]. Biotic processes in sediments can disproportionately remove N via denitrification [15]. Finally, sediment can also be a source of nutrients, delaying recovery from eutrophication [6].

Our study seeks to understand effects of fertilizer N:P ratio, and short-term recovery from fertilization, in a shallow pond in the species-rich subtropical desert valley of the Cuatro Ciénegas Basin (CCB) in Coahuila, Mexico. Located in the Chihuahuan desert, CCB has been coined a ‘biodiversity oasis’ and hosts a large number of endemic species, both in the macro- and microbiota [16]. CCB contains numerous spring-fed pools emptied by outflow streams that often lead to evaporative ponds and lagunas. These ponds and lagunas are shallow and often have molar TN:TP ratio of > 100, consistent with evidence of strong P limitation in a previous study of CCB’s stromatolites [17]. The genomes from microbialites in CCB are also enriched with genes encoding phosphorus transporters [18]. P-limitation also reduces horizontal gene transfer events by decreasing physical contact among cells, interaction with virus and enhanced scavenging of free DNA. In fact, it is hypothesized that the high microbial biodiversity found in this basin is driven by P-limitation itself [19]. The near-pristine springs and ponds in this area are a major tourist attraction for the town of Cuatro Ciénegas but concerns about agricultural expansion in the region are growing, as this threatens the regional aquifer [20]. Thus, gaining a better understanding of nutrient impacts on the ecosystems of CCB is particularly important.

This study describes the first effort to characterize the effects of nutrient enrichment in the Cuatro Ciénegas Basin’s shallow ponds. We determined the allocation of major nutrients between the sediment and water column and assessed the effects of nutrient stoichiometry on phytoplankton biomass and C:N:P stoichiometry. The experiment involved mesocosms that isolated 12.6-m^2^ sections of the water column and underlying sediment that were periodically fertilized with P alone or in combination with N at two N:P ratios (16 and 75) for three weeks. The mesocosms were also monitored for two weeks after fertilization ceased to determine the resilience of the pond to nutrient perturbation. We predicted that planktonic growth will be more responsive to nutrient enrichment with lower N:P ratio and that planktonic biomass elemental stoichiometry will change according to the enrichment N:P ratio. With minimal macrophytes and shallow water column, we expected the added nutrients to accumulate in the sediment after fertilization was ceased while the planktonic community returned to its initial state.

## Materials and Methods

### Study site

A replicated mesocosm experiment was conducted *in situ* in Lagunita (latitude: 26° 50’53.19”N, longitude: 102° 8’29.98” W), a shallow pond adjacent to a larger lagoon (Laguna Intermedia) in the Churince flow system. The physical and chemical characteristics of Lagunita prior to mesocosm installation are described in Table 1. The Churince flow system is located in the western region of the Cuatro Ciénegas basin in Coahuila, México. Consistent with Churince's hydrology, the water in Lagunita is high in conductivity and dominated by Ca^2+^, SO_4_^2-^, and CO_3_^2-^ (Table 1, [21]). Lagunita experiences strong evaporation, which decreased the water column depth from an average of 26 cm to 20 cm by day 21. A major rain event between days 21 and days 42 offset the evaporation effect, leaving a water column depth of 18 cm by the end of the experiment (Table 1). The N:P stoichiometry of the pond was highly imbalanced, with an average molar TN:TP ratio of 122 ± 22 observed during the 7-week study.

**Table 1:** Physico-chemical characteristics of Lagunita prior to mesocosm installation in May 2011 (day 0), after 3-weeks of fertilizer application (day 21) and 3-weeks after fertilizer application was ceased (day 42).

Parameters	Day 0	Day 21	Day 42
Water column depth (cm)^a^	20–32	13–27	12–26
Temperature (°C)^b^	21.5–39.7		
pH^c^	7.96	8.18	8.20
Conductivity (mS cm^-1^)^c^	9.17	11.50	9.40
Salinity (ppt)^c^	5.2	6.9	5.8
Chlorophyll *a* (*μ*g L^-1^)	15.3 ± 1.16	6.91 ± 0.98	31.3 ± 3.29
DOC	2177 ± 51.84	3578 ± 54.84	5787 ± 236
Nitrate/Nitrite	1.67 ± 0.34	0.98 ± 0.21	1.70 ± 0.60
Ammonia-N	1.07 ± 0.10	0.76 ± 0.05	0.88 ± 0.38
SRP	0.06 ± 0.02	0.27 ± 0.03	0.25 ± 0.07
TDN	130 ± 5.98	224 ± 6.39	309 ± 4.48
TDP	0.58 ± 0.17	1.16 ± 0.34	1.22 ± 0.26
Seston C	498 ± 34.45	778 ± 37.19	778 ± 42.64
Seston N	57.1 ± 2.81	93.9 ± 4.32	96.5 ± 8.21
Seston P	1.21 ± 0.08	1.77 ± 0.19	2.25 ± 0.13
Total N	187 ± 8.58	318 ± 8.52	405 ± 10.4
Total P	1.79 ± 0.20	2.93 ± 0.34	3.47 ± 0.29
Calcium ions (mmol L^-1^)	17.9 ± 1.15	17.5 ± 0.37	15.4 ± 0.61
Sulfate ions (mmol L^-1^)	486 ± 63.44	678 ± 18.76	956 ± 62.29
Sediment C (g C kg^-1^ sed)	87.5 ± 1.39	99.4 ± 0.33	N.D.
Sediment N (mmol N kg^-1^ sed)	248 ± 54.3	264 ± 33.5	213 ± 41.1
Sediment P (mmol P kg^-1^ sed)	1.48 ± 0.20	1.28 ± 0.10	N. D.

Values indicate the average of five measurements along the east-west transect ± 1 standard deviation. All values are in *μ*mol L^-1^ unless otherwise stated. DOC: Dissolved organic carbon, SRP: Soluble reactive phosphorus, TDN: Total dissolved nitrogen, TDP: Total dissolved phosphorus. N.D.; Not determined.

^a^ Range from five location.

^b^ Range obtained from 24 hours continuous temperature loggers.

^c^ No replicate readings were taken.

### Experimental design

The mesocosm experiment was conducted in the summer of 2011. Each mesocosm consisted of a thin round clear plastic tube with a diameter of 40 cm. The tube was pushed into the sediment to a depth of 20 cm and extended approximately 20 cm above the water surface. The mesocosms were arranged within the pond in a randomized complete block design with a total of 5 blocks. Each block consisted of four mesocosms, one from each treatment, with blocks deployed at the center of the pond along an east-to-west transect. Fish and larger aquatic macroinvertebrates were removed with a dip net prior to fertilization. Light intensity and temperature in the mesocosms did not differ from the pond (data not shown). In addition to an unenriched control treatment (“U” hereafter), the enrichment treatments were: P: amended with KH_2_PO_4_ to maintain a soluble reactive phosphorus (SRP) concentration at 1 *μ*mol L^-1^; NP16: as for P, but also amended with NH_4_NO_3_ at molar N:P ratio = 16; and NP75: as for P, but also amended with N at molar N:P ratio = 75. The experiment started with application of 1 *μ*mol L^-1^ P and the corresponding N into each mesocosm on day 0. The SRP concentration of each mesocosm was measured every 3–4 days (see *Chemical Analysis*), followed by the addition of KH_2_PO_4_ required to bring its *in situ* concentration back up to 1 *μ*mol L^-1^. The amount of NH_4_NO_3_ added into each mesocosm was calculated based on the predetermined N:P ratio for that treatment. Fertilizers were added by pipetting nutrient solutions into the mesocosms and then mixing with a rinsed dip net. Nothing was added to the U mesocosms but these were stirred at the time of fertilization to mimic mixing in the enriched mesocosms. Fertilizer application was continued for 3 weeks until day 21.

### Mesocosm sampling

All sampling was conducted from boardwalks suspended from the shore to minimize disturbance to the pond. Physical measurements (pH, temperature, conductivity and water column depth), salinity and dissolved oxygen were measured prior to water and sediment collection using a YSI Model 85 meter (Yellow Springs Instruments Inc., Yellow Springs, OH) and Beckman Coulter 255 pH/mV meter (Beckman Coulter Inc., Brea, CA). Water collected prior to fertilizer application and every 3–4 days thereafter for SRP quantification was filtered in the field through 1.2-*μ*m polyethersulfone membrane filters (Pall Life Sciences, Port Washington, NY) and transported to the field lab on ice. SRP was then measured using the ascorbic acid colorimetric assay within 2 hours of sampling [22].

Extensive sampling of water and sediment for chemical and biological parameters was conducted on day 21, which was after 3 weeks of periodic fertilization; and on day 42, which was after 2.5 weeks the fertilization was ceased. Water samples were also collected on day 6 for quantitation of chlorophyll *a* (Chl *a*). Water was collected in acid-washed 2-L cubitainers while sediment was sampled by scraping the top 2 mm of sediment with a sample dipper. Sediment was homogenized and transferred into cryovials. Both water and sediment were transported to the field lab on ice.

Water samples were filtered through pre-combusted (24 h at 450°C) GF/F filters (0.7μm) and GF/C filters (1.2 μm, Whatman, Piscataway, NJ) for seston elemental analysis and Chl *a* quantitation, respectively, and stored at -20°C. To measure total dissolved nutrients, water samples were filtered through 0.2-*μ*m polyethersulfone membrane filters. Samples for dissolved organic carbon (DOC), and total dissolved nitrogen (TDN) were acidified with 12N HCl to pH < 2 and stored in the dark at room temperature, while the remaining filtrate was frozen for SRP, total dissolved P (TDP), total ammonia (NH_3_/NH_4_^+^), nitrate (NO_3_^-^), and nitrite (NO_2_^-^) analyses. Sediment samples for nutrient analyses were stored at -20°C. Sediment for biomass elemental composition was flash-frozen with liquid nitrogen in 15% glycerol and stored at -80°C.

### Chemical analyses

GF/F filters with seston were thawed, dried at 60°C and then packed into tin discs (Elemental Microanalysis, U.K.) for C and N analyses with a Perkin Elmer PE 2400 CHN Analyzer at the Arizona State University Goldwater Environmental Laboratory (ASU GEL). Another set of dried GF/F filters prepared from the same water samples was used for estimating seston P content. These filters were digested in persulfate followed by a colorimetric analysis to determine PO_4_^3-^ [23]. Chl *a* was quantified fluorometrically using a TD-700 fluorometer (Turner Designs, Sunnyvale, CA) after 16–24 hours of extraction in cold absolute methanol [24].

TDP concentrations were determined using the colorimetric assay after persulfate digestion as described above; SRP was measured without the persulfate digestion step. DOC and TDN were analyzed using the Shimadzu TOC-VC/TN analyzer at the ASU GEL. Nitrate was reduced to nitrite and then quantified using a Lachat QC8000 Flow Injection Analyzer. Total ammonia-N (NH_3_/NH_4_^+^) was quantified using the orthophthaldialdehyde (OPA)-ammonium based fluorometric method [25]. Total N (TN) and total P (TP) concentrations were calculated as the sum of the seston and total dissolved pools.

Sediment samples were thawed and dried at 60°C prior to analyses. For C and N analyses, the dried sediments were packed into tin capsules for analysis with the PE 2400 CHN Analyzer as described above. For P quantitation, sediment samples were digested in persulfate followed by colorimetric assay as described above. To measure elemental composition of sediment microbes, cells were first extracted from sediment using a combination of chemical treatment with the non-ionic detergent Tween 20 and sodium pyrophosphate, and physical treatment by sonication to break cell-sediment bonds [26]. The cells were then purified by Nycodenz (Axis-Shield, Norway) density gradient centrifugation. Separated cells were dried at 60°C and weighed prior to analysis by mass spectrometry. A fraction of the recovered biomass was combusted to measure C and N from CO_2_ and N_2_ using an Isotope Ratio—Mass Spectrometer (IRMS; Costech, Italy). The rest was digested using concentrated HNO_3_ at 100 to 150°C overnight, prior to analysis for P by Inductively Coupled Plasma—Mass Spectrometry (ICP-MS; Thermo iCap Q, Thermo Fisher Scientific, USA). A minimum dry mass of approximately 1 mg was necessary to yield quantitative IRMS measurements (i.e., within the calibration curve). When a given separation clearly did not yield enough cell material to carry out analyses on several biological replicates, we combined the cells of replicate separations. Thus, two combined replicates were analysed for treatments U, P, and NP75; while 3 combined replicates were analysed for treatment NP16.

Blanks were carried through the digestion and analysed along with the samples. To ensure that microbial elemental content was not altered during the procedure, wild-type *E*. *coli* of known elemental composition [26] was separated and analysed along with each sample. During elemental analyses, a calibration curve was built using standards of known elemental composition. The accuracy of analyses was verified using check standards analysed along with the samples. Elemental composition of the control *E*. *coli* culture and validation of the method was described in detail by Neveu et al., 2014 [26]. Analysis of elemental composition of the separated cells assumes that sediment contamination was negligible. Considering a worst-case scenario, where the separated cells were contaminated with calcite (12% C by mass) the separated material will consist of 50% calcite and 50% cells. However, the Ca^2+^ to CO_3_^-^ ratio in the Churince system is much higher than 1:1 [21]. In addition to that, other compounds such as Mg^2+^ and SO_4_^2-^ are also present in high concentrations in Churince sediment. Hence, it was inferred that sediment contamination in the separated material was insignificant. The contamination will also not affect changes in C:N:P ratio among the different treatments since the sediment characteristics across all treatments were the same.

### Data analyses

All elemental ratios are presented as atomic ratios. Randomized-block ANOVA was performed for data collected on days 6, 21 and 42 separately to determine the statistical significance of fertilization effects. Pairwise comparisons for significant treatment differences were evaluated using the *post hoc* Tukey’s test (p ≤ 0.05). Resilience of the pond to the fertilization was determined using repeated measures ANOVA on data collected from both days 21 and 42. If a significant time effect was detected, a Student’s *t*-test was conducted for each treatment pair. All statistical analyses were performed using the multcomp package in R [27]. Outliers beyond three standard deviations from the mean were omitted from statistical analyses, specifically for nitrate and ammonia data. Removal of outliers did not affect the statistical outcome.

## Results

### Fertilizer application

All fertilized treatments received soluble inorganic P in the form of KH_2_PO_4_ repeatedly for 21 days. By day 21, a total of 154–161 *μ*moles of P had been added to each fertilized mesocosm. Based on this level of addition, P fertilization was expected to increase the water column total phosphorus (TP) in fertilized treatments by more than 3.5-fold (from 1.8 to 6.7 *μ*mol L^-1^). NP16 and NP75 treatments also received a total of 2.5 mmoles and 12.1 mmoles of N, respectively. Assuming all N was retained in the water column, the NP16 treatment was expected to have 40% more nitrogen in the water column than the U treatment (an increase from 236 to 336 *μ*mol L^-1^) while NP75 treatment should experienced a 3-fold increase in water column total nitrogen (TN, an increase from 236 to 726 *μ*mol L^-1^). Fertilizer application had no effect on physical characteristics except pH. The NP75 treatment had significantly higher pH at 8.1 ± 0.18 compared to the U treatment (7.9 ± 0.17) on day 21 but decreased back to the U treatment level when fertilizer application was ceased.

### Initial response period (3 weeks after periodic fertilization)

#### Phosphorus concentrations

Soluble reactive phosphorus (SRP) concentrations in all fertilized treatments (P, NP16, NP75) after 3 weeks of periodic fertilization were either lower or similar to the SRP concentration in the unenriched treatment (F_3,12_ = 5.58, p-value = 0.012; Table 2). The low SRP concentrations in enriched treatments indicated that phosphate was rapidly removed from the water column, with P most efficiently removed in the NP75 treatment. Consequently, the TDP in enriched treatments was also similar to or lower than TDP in the unenriched treatment (F_3,12_ = 8.67, p-value = 0.002, Table 2). Using seston as a proxy for planktonic biomass, approximately one third of the applied P (154–161 μmoles) in all fertilized treatments was immobilized into the seston pool. Seston P in all fertilized treatments more than doubled (F_3,12_ = 28.35, p-value < 0.001) and TP significantly increased (F_3,12_ = 15.86, p-value < 0.001; Table 2). In addition, a significant increase in sediment P was also observed for the enriched treatments (F_3,9_ = 10.32, p-value = 0.003, Table 2). The increase in surface sediment P content suggests that 36% of the added phosphate was sequestered into the top 2 mm of surface sediment.

**Table 2:** Phosphorus pools in the mesocosms on day 21 and 42 for the four treatments.

	SRP		TDP		Seston P		Total P		Sediment P	
	(*μ*mol L^-1^)		(*μ*mol L^-1^)		(*μ*mol L^-1^)		(*μ*mol L^-1^)		(mmol kg^-1^)	
Treatment	D21	D42	D21	D42	D21	D42	D21	D42	D21	D42
U	0.31 **a**	0.30	1.39 **a**	1.40	1.09 **a**	1.06 **a**	2.47 **a**	2.46 **a**	1.16 **a**	1.23 **a**
	(0.09)	(0.05)	(0.25)	(0.10)	(0.11)	(0.22)	(0.24)	(0.32)	(0.20)	(0.28)
P	0.33 **a**	0.36	1.24 **a**	1.56	2.71 **b**	1.77 * **bc**	3.95 **b**	3.34 **bc**	1.44 **b**	1.71 **b**
	(0.10)	(0.14)	(0.28)	(0.15)	(0.33)	(0.23)	(0.59)	(0.27)	(0.30)	(0.18)
NP16	0.27 **ab**	0.33	1.15 **ab**	1.38	3.03 **b**	1.64 * **b**	4.17 **b**	3.03 * **b**	1.47 **b**	1.54 **b**
	(0.12)	(0.05)	(0.40)	(0.09)	(0.26)	(0.12)	(0.62)	(0.13)	(0.16)	(0.20)
NP75	0.19 **b**	0.35 *	0.86 **b**	1.49 *	3.29 **b**	2.16 * **c**	4.15 **b**	3.64 **c**	1.65 **b**	1.61 **b**
	(0.08)	(0.08)	(0.25)	(0.25)	(0.68)	(0.49)	(0.71)	(0.34)	(0.15)	(0.08)

Each value represents the average and one standard deviation in parentheses. Different bolded letters indicate significant differences between the treatments within day, based on randomized block ANOVA.

* on day 42 denotes a significant change from day 21 based on Student’s t-test.

#### Nitrogen concentrations

**Fig 1:**
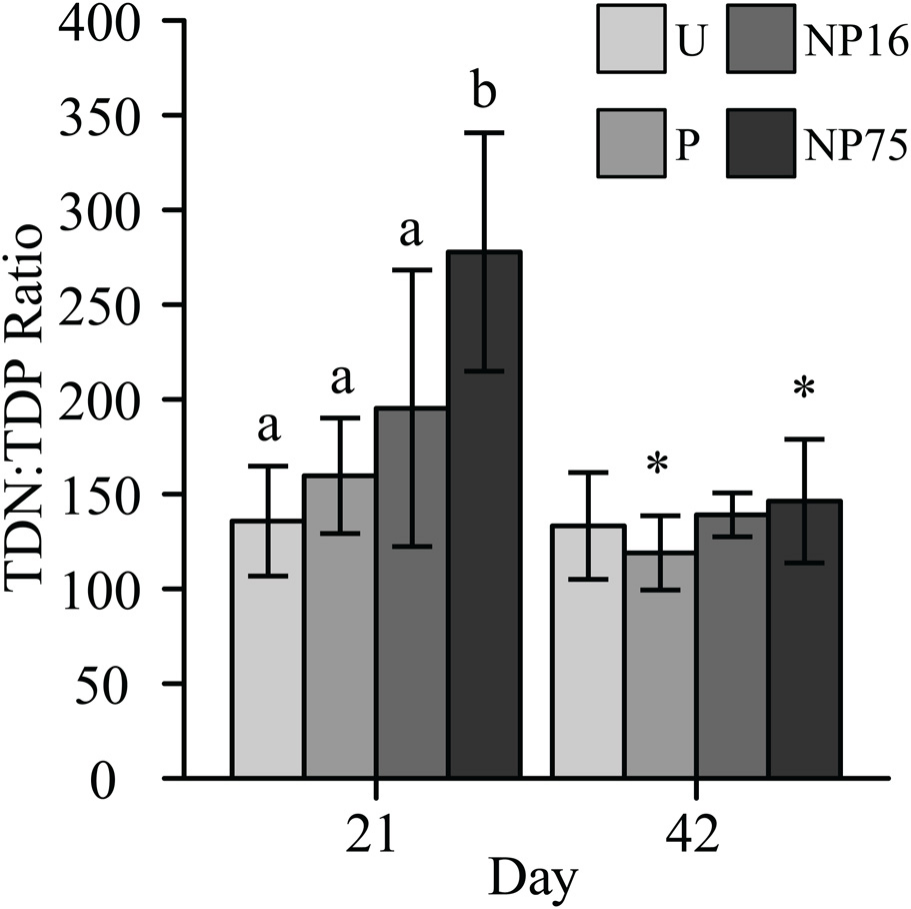
N:P ratio of residual dissolved nutrients in the water column on days 21 and 42. Each bar represents average of 5 measurements ± 1 standard deviation. Different letters indicate significant differences between treatments for a given date while ‘*’ represents a significant change from day 21.

Dissolved N concentrations in the fertilized treatments depended on the N:P ratio of the added nutrients. NO_3_^-^/NO_2_^-^ concentrations in P and NP16 treatments were as low as concentrations in the unenriched treatment, but the NP75 treatment had a significantly higher concentration (F_3,11_ = 4.98, p-value = 0.02, Table 3). Notably, ammonia-N in the unenriched mesocosms accumulated to concentrations of approximately 2-fold higher than in the pond, which remained at 1 μM or less throughout the experiment (Tables 1 and 3). Phosphorus enrichment in the P and NP16 treatments alleviated this accumulation, decreasing ammonia levels to lower than in the pond (F_3,10_ = 14.85, p-value < 0.001; Table 3). Total ammonia concentration in the NP75 treatment was intermediate at half the concentration in unenriched treatment (Table 3). The differential response of dissolved N species to N:P ratio of applied fertilizer was also evidenced by the significantly increased TDN concentration in the NP75 treatment only (F_3,12_ = 10.73, p-value < 0.001, Table 3). Furthermore, residual dissolved nutrients in NP75 had significantly higher N:P ratio (F_3,12_ = 12.74, p-value < 0.001; Fig 1).

**Table 3:** Nitrogen pools in the mesocosms on day 21 and 42 for the four treatments.

	NO_3_^-^/NO_2_^-^		NH_3_/NH_4_^+^		TDN		Seston N		Total N		Sediment N^a^	
	(*μ*mol L^-1^)		(*μ*mol L^-1^)		(*μ*mol L^-1^)		(*μ*mol L^-1^)		(*μ*mol L^-1^)		(mmol kg^-1^)	
Treatment	D21	D42	D21	D42	D21	D42	D21	D42	D21	D42	D21	D42
U	0.96 **ab**	1.40	2.33 **a**	16.33	183 **a**	185 **ab**	53.0 **a**	49.8 **a**	236 **a**	235 **a**	182	242
	(0.20)	(0.47)	(0.64)	(12.51)	(9.51)	(32.81)	(3.73)	(21.44)	(9.60)	(42.32)		(17)
P	0.65 **a**	1.15	0.16 **b**	4.13	192 **a**	184 **a**	76.5 **a**	76.3 **ab**	269 **a**	260 **a**	212	243
	(0.24)	(0.77)	(0.15)	(3.75)	(17.88)	(20.81)	(14.05)	(12.77)	(31.49)	(32.81)		(74)
NP16	0.89 **ab**	1.01	0.37 **b**	3.41 *	202 **a**	192 **ab**	110 **b**	84.2 **ab**	312 **b**	276 **ab**	186	257
	(0.18)	(0.39)	(0.10)	(2.44)	(12.11)	(16.08)	(23.78)	(20.07)	(34.04)	(33.67)		(27)
NP75	2.89 **b**	1.38	1.21 **ab**	3.59	226 **b**	212 **b**	117 **b**	103 **b**	340 **b**	315 **b**	247	ND
	(2.04)	(0.52)	(0.73)	(2.14)	(25.63)	(23.87)	(14.91)	(37.57)	(16.27)	(53.90)		

Each value represents the average and one standard deviation in parentheses. Different bolded letters indicate significant differences between the treatments within day, based on randomized block ANOVA. ND: Not determined.

* on day 42 denotes a significant change from day 21 based on Student’s t-test.

^a^ No replication was made for this analysis due to insufficient sediment samples.

As expected, P only application had a limited effect on seston N while simultaneous N and P application in the NP16 and NP75 treatments resulted in more than a two-fold increase in seston N (F_3,11_ = 14.61, p-value < 0.001). Consequently, a significant increase in total N (F_3,11_ = 24.93, p-value < 0.001) concentrations in the water column was also observed in NP16 and NP75 treatments (Table 3). In the NP16 treatment, an average of 76% of the applied N was found in the water column with 57% of the applied N immobilized into seston. While seston N and TN concentrations in the NP75 treatment were not significantly different from those in NP16, only 13% of the applied N in the NP75 treatment could be accounted for in the water column. Sediment N content was slightly elevated in the NP75 treatment when compared to the U treatment (Table 3). However, it could not be determined if the difference was statistically significant because of incomplete data due to insufficient sediment samples for N analysis.

#### Carbon and plankton response

**Fig 2:**
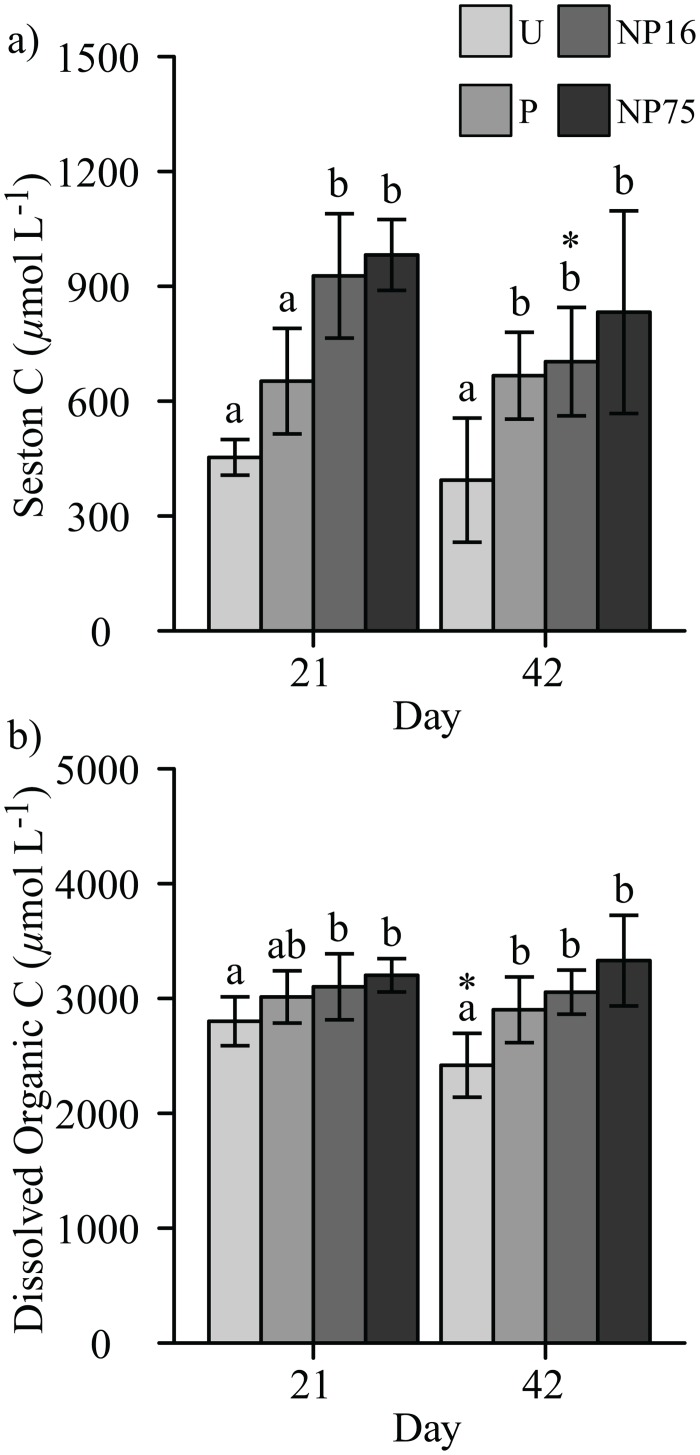
Effects of fertilizer application on a) seston C and b) dissolved organic carbon, and their recovery. Each bar represents average of 5 measurements ± 1 standard deviation. Different letters indicate significant difference between treatments for a given date while ‘*’ represents a significant change from day 21.

**Fig 3:**
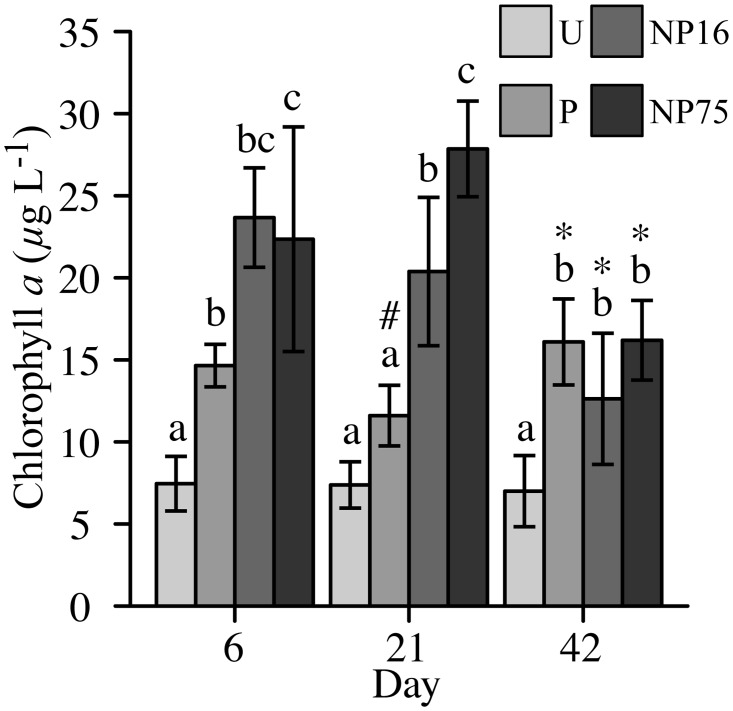
Water column chlorophyll *a* concentrations during fertilizer application (days 6 and 21) and after cessation of fertilizer application (day 42). Each bar represents average of 5 measurements ± 1 standard deviation. Different letters indicate a significant difference between treatments for a given date. Symbol ‘#’ represents a significant change from day 6 while ‘*’ represents a significant change from day 21.

Increased concentrations of dissolved organic C (DOC) and seston C were observed in the simultaneous N and P enriched treatments (F_3,12_ = 7.24, p-value = 0.005, Fig 2). Nutrient enrichment also increased Chl *a* concentration, which was apparent as early as 6 days after fertilizer application (F_3,10_ = 21.87, p-value < 0.001, Fig 3). However, on day 21, elevated Chl *a* was only observed for the NP16 and NP75 treatments (F_3,12_ = 53.78, p-value < 0.001, Fig 3). Increased C in seston was consistent with increased phytoplankton biomass as indexed by Chl *a* concentration (F_3,11_ = 21.09, p-value < 0.001, Figs 2a and 3). Nevertheless, no correlation between Chl *a* concentration or seston C concentration and N:P ratio was observed.

While P enrichment alone had only marginal effects on seston C concentration, it did significantly decrease seston C:P ratio (F_3,11_ = 16.57, p-value < 0.001) and N:P ratio (F_3,11_ = 11.03, p-value = 0.001; Table 4). Similar decreases in seston C:P and N:P ratios were also observed for the NP16 and NP75 treatments (Table 4). Nutrient enrichment did not change the seston C:N ratio.

**Table 4:** Elemental stoichiometry of seston on days 21 and 42 in the mesocosms.

	C:N		C:P		N:P	
Treatment	D21	D42	D21	D42	D21	D42
U	8.55	7.96	419.9 **a**	366.9	49.12 **a**	46.29
	(0.64)	(0.32)	(56.8)	(106.9)	(6.04)	(14.25)
P	8.51	8.77	240.1 **b**	390.8 *	28.26 **b**	44.98 *
	(0.39)	(0.99)	(29.5)	(23.9)	(3.75)	(4.40)
NP16	8.46	8.42	305.2 **b**	427.2 *	36.39 **b**	51.07 *
	(0.63)	(0.74)	(36.2)	(74.1)	(6.34)	(10.52)
NP75	8.47	8.20	306.8 **b**	381.7	36.25 **b**	46.64
	(0.44)	(0.82)	(53.1)	(82.0)	(6.20)	(8.91)

Each value represents the average and one standard deviation in parentheses. Different bolded letters indicate significant different between the treatments within the same day.

* denotes significant change from day 21.

#### Sediment microbes

**Fig 4:**
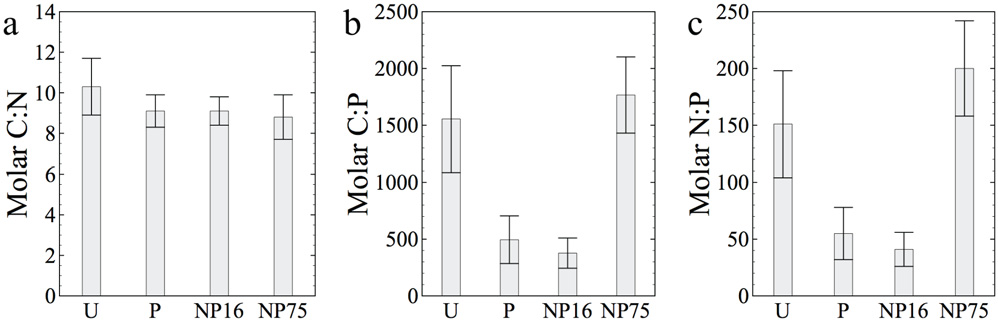
Effects of fertilizer application on sediment microbe biomass (separated cells) a) C:N, b) C:P, and c) N:P molar ratios for samples collected on day 21. Each bar represents the average of two measurements (three for NP16 treatment) ± 1 standard deviation.

The atomic C:N, C:P, and N:P ratios of sediment microbes for each of the four treatments on day 21 are shown in Fig 4. Sediment microbes in Lagunita had extremely high C:P and N:P ratios (Fig 4b and 4c). P enrichment in the P and NP16 treatments resulted in a four-fold decrease for both C:P and N:P in microbial biomass. However, microbial C:P and N:P ratios in the NP75 treatment were similar to those in the control (U). C:N ratios of microbe cells were unchanged across treatments.

### Recovery period (2.5 weeks after last fertilizer application)

#### Phosphorus concentrations

After 2.5 weeks of recovery, SRP and TDP concentrations in the NP75 treatment returned to levels observed in the U treatment (Table 2). Seston P concentrations in the three fertilized treatments decreased significantly from day 21 but remained elevated compared to the U treatment (F_3,11_ = 15.30, p-value < 0.001; Table 2). A similar observation was made for water column TP (F_3,11_ = 16.81, p-value < 0.001, Table 2). As observed on day 21, sediment P concentrations remained elevated in all the fertilized treatments (F_3,11_ = 9.58, p-value = 0.002, Table 2).

#### Nitrogen concentrations

Nitrate concentration in the NP75 treatment decreased from day 21 to levels seen in the other treatments (Table 3). Total ammonia concentration in the U treatment continued to increase, reaching values 2- to 11-fold higher than values on day 21 (Table 3). Total ammonia also increased in the fertilized treatments when compared to day 21, albeit at a much slower rate than in the U treatment (Table 3). The amount of NH_3_/NH_4_^+^ accumulated in all treatments was highly variable, resulting in large standard deviations (Table 3). The difference in TDN concentration between the four treatments had also decreased when compared to day 21 (Table 3). Consequently, the TDN:TDP ratio of residual nutrients in the NP75 treatment significantly decreased down to values in the range of the U treatment (Fig 1). Seston N (F_3,12_ = 5.71, p-value = 0.011) and TN (F_3,12_ = 7.79, p-value = 0.004) concentrations in the NP16 treatment decreased slightly towards values similar to those in the U treatment (Table 3). However, the NP75 treatment continued to have elevated TN and seston N (Table 3).

#### Carbon and phytoplankton response

All fertilized treatments continued to have Chl *a* concentrations significantly higher than the U treatment after fertilizer application was ceased (F_3,12_ = 11.90, p-value = 0.001, Fig 3). Carbon concentration in the seston pool for NP16 decreased slightly, but all fertilized treatments maintained elevated C concentrations in seston when compared to the U treatment (F_3,12_ = 8.17, p-value = 0.003, Fig 2a). A similar observation was made for water column DOC concentrations (F_3,12_ = 11.06, p-value = 0.001, Fig 2b). Indeed, the difference in DOC between the U and fertilized treatments was enhanced by a significant decrease in DOC concentration in the U treatment from day 21 to day 42 (Fig 2b). On the other hand, seston C:P and N:P ratios in fertilized treatments increased to values similar to those observed in the U treatment after fertilizer application was ceased (Table 4).

## Discussion

In this study, the effect of nutrient loading and fertilizer N:P ratio on a shallow pond ecosystem in the remote CCB was assessed through a replicated *in situ* mesocosm experiment. As expected due to its relatively low nutrient concentrations and highly imbalanced stoichiometry (high N:P ratio), Lagunita was highly sensitive to P enrichment. Enrichment effects were apparent within days of initial fertilization (Fig 3). Added PO_4_ was actively immobilized into seston (Table 2), supporting the expectation that Lagunita is P-limited due to its high TN:TP ratios. However, addition of P alone only had significant impacts on Chl *a* concentrations and seston elemental ratios (Fig 3, Table 4) and simultaneous N and P enrichment resulted in a considerably larger response of planktonic biomass (Fig 2a). Furthermore, the elevated Chl *a* concentration observed on day 6 in the P-only treatment could not be sustained by further addition of P, suggesting that P was not the only limiting nutrient (Fig 3). While combined N and P enrichments have often been found to produce a larger phytoplankton growth response than P-only enrichment [1, 28], this observation was unexpected in Lagunita given its pronounced N:P imbalance (TN:TP = 122 ± 21, TDN:TDP = 254 ± 90; by atoms). Furthermore, the amount of N added in the NP16 treatment represented only a 40% increase in TN while P addition increased TP by 3-fold and thus was expected to yield a larger effect.

Nitrogen limitation of at least some of the microbial biota in Lagunita is also suggested by the depletion of NH_3_/NH_4_^+^ in the water column. A notable accumulation of NH_3_/NH_4_^+^ was observed for the unenriched treatment (relative to the pond itself) while the P and NP16 treatments had concentrations lower than the pond's NH_3_/NH_4_^+^ concentration (Table 3). In fact, a majority of the added N was sequestered into the seston pool, accounting for 76% of the added N in the NP16 treatment. We note that a large fraction of dissolved N in Lagunita was in the dissolved organic form (DON), calculated as the difference between total dissolved N and dissolved inorganic N pools (Table 1). Although DON can be a source of N to bacterioplankton, some forms of DON can be recalcitrant and require more energy to assimilate than NO_3_^-^ or the highly reduced NH_4_^+^ [29]. The potential unavailability of DON has led to the suggestion that dissolved inorganic nitrogen (DIN):TP or DIN:TDP ratios can serve as a better predictor for nutrient limitation in oligotrophic lakes [8]. Indeed, the DIN:TP (~1 by atoms) and DIN:TDP (3.07 ± 1.5, by atoms) ratios in Lagunita are consistent with strong N limitation according to the regression analysis approach of Bergstrom [30]. Therefore, it is still very likely that at least some microbiota in Lagunita experience N limitation along with others experiencing P limitation, despite the pond's high TN:TP ratio [8]. These observations for Lagunita stress the need to consider both nitrogen and phosphorus dynamics when studying nutrient limitation in freshwater ecosystems [1, 11, 28] and provide further caution against relying on TN:TP ratios to infer nutrient limitation in the absence of experimental data.

The response to P enrichment was more apparent in the stoichiometric composition of the seston than it was in the biomass of seston. This result is similar to that seen in mesocosm studies performed in P-limited Lake 239 at the Experimental Lakes Area, where P addition marginally increased seston C concentration but produced strong decreases in seston C:P and N:P ratios [31]. The change in biomass stoichiometry was mainly driven by P because both NP16 and NP75 treatments had seston with similar elemental ratios (Table 4). In the NP75 treatment, the high fertilizer N:P ratio retained a primarily P-limited condition. The excess N may have been denitrified rather than assimilated as biomass. In fact, only 20% of the added N in the NP75 treatment can be accounted for, with most of it in the seston pool. Denitrification genes have been found in a nearby ecosystem [32] and their abundance is currently being explored in metagenomes generated from the pond.

Lagunita's responses are also consistent with previous observations of strong decreases in C:P ratios of stromatolitic biomass in Rio Mesquites, another aquatic ecosystem in CCB, in response to P enrichment [17]. Nonetheless, both seston and sediment biomass C:P and N:P ratios are still very much higher than the more commonly reported Redfield proportions of 106 (C:P) and 16 (N:P) and still exceed a threshold value thought to be generally indicative of a transition between N and P limitation (N:P ~31:1 by atoms) [33]. Overall, these observations are consistent with studies that show considerable variation of seston C:P and N:P ratios in freshwater ecosystems due to environmental conditions of nutrient supply and other factors [34, 35]. The dependence of seston C:N:P ratio on the fertilizer application suggests that the planktonic community has adapted to the high N:P ratio of available nutrients at the Cuatro Ciénegas aquatic ecosystems.

Effects of nutrient amendment in shallow water bodies can be dampened due to sequestration of added nutrients into sediments [13]. This sequestration can involve biological uptake as well as chemical processes. Biological uptake plays a significant role for shallow lakes where the benthic surface layers are dominated by macrophytes and photoautotrophic microorganisms. In fact, nutrient additions may be completely assimilated by the benthic community and have a minimal effect on planktonic community [36]. Lagunita largely lacks macrophytes and thus chemical sequestration may have been more important. The sediment pore waters in Lagunita have relatively high pH (>7), and high concentrations of calcium (15–17 mmol/L) and carbonates [21]. High concentrations of calcium and carbonate ions may favour potential co-precipitation and trapping of nutrients into calcite precipitates or microbialites [14, 18]. Based on the sediment dry weight and P content, we estimated that approximately 36% of the added P was adsorbed to the top 2 mm of the sediment surface, indicating considerable capacity for sediment P adsorption to buffer against the increased external nutrient loading. Nevertheless, there was still a positive response of planktonic biomass to fertilization, especially for the NP16 and NP75 treatments (Fig 2a). This rapid uptake of added nutrients by the plankton before potential sequestration in sediments is consistent with strong nutrient limitation in Lagunita. Biological nutrient uptake is not surprising given that the entire water column is well aerated and within the photic zone, providing abundant carbon and energy sources for photoautotrophic production. With another 30% of the P immobilized into the seston pool, the remaining one third of the added P could not be accounted for in the fertilized treatments. It is hypothesized that the remaining P added into the mesocosms found its way into the deeper sediments, which are relatively unconsolidated up to at least 90 cm depth. The large amount of pore water and high ion concentrations can promote calcite precipitation and interaction of P with other metals such as Fe and Mg^2+^ [14].

Although the added nutrients were mostly consumed by the planktonic community, a significant change in the elemental stoichiometry of sediment microbes was nevertheless apparent (Fig 4). The P-only and NP treatment biomass C:P and N:P ratios drastically decreased to levels similar to the planktonic biomass (Fig 4). Unlike the planktonic biomass, sediment microbes in the NP75 treatment still had high C:P and N:P ratios (Fig 4) comparable to the unenriched treatment. This observation is consistent with the hypothesis that the NP75 mesocosm remained P-limited. Like the planktonic biomass, the sediment microbes still had highly skewed elemental ratios, indicating nutrient limitation. While it is common to find benthic microorganisms with elevated C:P ratios (higher than 106 but generally <1000:1, [37, 38]), sediment biomass in Lagunita had some of the highest C:P ratios reported for aquatic microorganisms (> 1500:1).

In some freshwater systems, the capacity of sediments to buffer against P inputs decreases over time as nutrients are increased [13]. Eventually, the sediments can become a source of P rather than a sink, especially after the external loading is removed [13]. To address this possibility, we monitored the recovery of Lagunita for two weeks after the last fertilizer application. Net internal loading of P was not observed since TP decreased for all fertilized treatments after enrichment was stopped (Table 2). Seston P in all the fertilized treatments also decreased significantly from day 21 (Table 2). Most importantly, sediment P in fertilized treatments remained elevated when compared to the unenriched sediment and did not change from day 21 (Table 2). Hence, the sediments of CCB appear to be important for buffering some of the external nutrient loading.

Recovery after cessation of fertilizer application was not as obvious for N as it was for P in the mesocosms. Seston N and TN in the NP16 and NP75 treatments slightly decreased, although not quite as low as concentrations in the unenriched mesocosms (Table 3). Unexpectedly, total ammonia concentrations developed to remarkably high concentrations by the end of the experiment (>16 *μ*mol L^-1^) in the unenriched treatment compared to the pond itself. Ammonia-N concentrations in the fertilized treatments accumulated at a slower rate, suggesting that fertilization initially kept ammonium concentrations low due to increased biological demand from the P enrichment, at least until the P supply ceased. Accumulation of ammonia-N concentration could be due to two reasons—the breakdown of DON into NH_4_^+^ or release of NH_4_^+^ from cell death. As previously mentioned, a large fraction of TDN in Lagunita is in the organic form. DON can undergo photochemical oxidation producing ammonium as the major product. This reaction occurs most efficiently at ultraviolet wavelengths [39], a condition highly likely in CCB where there is extensive exposure to solar radiation in the summer. DON can also be converted to NH_4_^+^ by ammonifying bacteria. Alternatively, cell lysis or decomposition of zooplankton can lead to NH_4_^+^ production. In the presence of the limiting nutrient, P, any NH_4_^+^ produced can be assimilated (P and NP16 treatment, Table 3). When the fertilizer application was ceased, the plankton slowly returned to the P-limitation state, allowing NH_4_^+^ to start accumulating. Since these ammonification processes are also occurring in the pond itself, the accumulation of NH_4_^+^ in the unenriched mesocosms relative to the pond requires additional explanation. We suspect that this accumulation may reflect differences in volatilization of ammonia (NH_3_), which becomes increasingly prevalent at high pH values such as those in Lagunita. That is, the walls of the enclosures, which extended at least 20 cm above the water surface, may have protected the water column in the enclosures from prevailing strong winds, decreasing rates of atmospheric transfer of NH_3_. This possibility awaits further investigation.

Increasing sources of nutrient loading from local and regional expansions of agriculture, atmospheric deposition, and human habitation are placing many seemingly pristine and remote water bodies at risk for eutrophication [4, 5], including those in the CCB. Increased nutrient inputs to CCB are of great concern because the valley hosts a large number of aquatic ecosystems where the organisms have adapted to low TP concentrations and imbalanced TN:TP ratios [40]. For example, microbial biomass of stromatolites in Rio Mesquites at CCB has C:P ratios of 750–2000 [17]. This study found that Lagunita, a shallow pond in the western arm of CCB, is highly sensitive and relatively unresilient to nutrient perturbation, despite the potentially strong capacity of its sediments to sequester nutrient inputs. While P enrichment stimulated short-term chlorophyll *a* production, its main impacts were in affecting the elemental composition of the microbial biomass, both in the water column and sediment surface. Thus, planktonic biomass responded relatively modestly to P-enrichment when added alone. Surprisingly given the very high TN:TP ratios in Lagunita, both the planktonic and sediment community nevertheless exhibit secondary nutrient limitation for N when P was supplied. While the main concern for CCB is focused on extensive water extraction for agriculture purposes [41], this study confirms that aquatic ecosystems at CCB are highly nutrient-limited and supports a view that their effective conservation will depend, at least in part, on protecting them from anthropogenic nutrient inputs, including both phosphorus and nitrogen.
